# Objective Assessment of Attention-Deficit Hyperactivity Disorder (ADHD) Using an Infinite Runner-Based Computer Game: A Pilot Study

**DOI:** 10.3390/brainsci10100716

**Published:** 2020-10-09

**Authors:** David Delgado-Gómez, Aaron Sújar, Juan Ardoy-Cuadros, Alejandro Bejarano-Gómez, David Aguado, Carolina Miguelez-Fernandez, Hilario Blasco-Fontecilla, Inmaculada Peñuelas-Calvo

**Affiliations:** 1 Department of Statistics, Universidad Carlos III, Getafe, 28903 Madrid, Spain; ddelgado@est-econ.uc3m.es (D.D.-G.); 100329944@alumnos.uc3m.es (A.B.-G.); 2 Department of Psychiatry, Puerta de Hierro University Hospital, Health Research Institute Puerta de Hierro-Segovia de Arana (IDIPHISA), 28222 Majadahonda, Spain; aaronsujar@idiphim.org (A.S.); hilario.blasco@salud.madrid.org (H.B.-F.); 3 Department of Psychology, Faculty of Health Sciences, University Rey Juan Carlos, Avda. Atenas s/n, 28922 Alcorcón, Madrid, Spain; 4 Department of Social Psychology and Methodology, Autonoma University of Madrid, 28040 Madrid, Spain; david.aguado@uam.es; 5 Instituto Ingeniería del Conocimiento, Autonoma University of Madrid, 28040 Madrid, Spain; 6 CIBERSAM, Autonoma University of Madrid, ITA-Mental Health, 28049 Madrid, Spain; carolina.miguelezfer@gmail.com; 7 Department of Child and Adolescent Psychiatry, Hospital Universitario Fundación Jiménez Díaz, 28040 Madrid, Spain; inmaculada.penuelas@quironsalud.es; 8 School of Psychology, Universidad Complutense de Madrid, Pozuelo de Alarcón, 28223 Madrid, Spain

**Keywords:** ADHD, video games, inattention, SWAN, e-health

## Abstract

In the last few years, several computerized tasks have been developed to increase the objectivity of the diagnosis of attention-deficit hyperactivity disorder (ADHD). This article proposes the “running raccoon” video game to assess the severity of inattention in patients diagnosed with ADHD. Unlike existing tests, the proposed tool is a genuine video game in which the patient must make a raccoon avatar jump to avoid falling into different gaps. The distance to the gap is recorded for each jump. To evaluate the proposed game, an experiment was conducted in which 32 children diagnosed with ADHD participated. For each participant, the median and interquartile range of these distances were calculated, along with the number of omissions. Experimental results showed a significant correlation between the participants’ inattention (measured by the Attention-Deficit/Hyperactivity Disorder Symptoms and Normal Behavior rating scale (SWAN) inattention subscale) with each of these three measures. In addition to its accuracy, other benefits are its short duration and the possibility of being run on both standard computers and mobile devices. These characteristics facilitate its acceptance in clinical environments or even its telematic use. The obtained results, together with the characteristics of the video game, make it an excellent tool to support clinicians in the diagnosis of ADHD.

## 1. Introduction

Attention-deficit hyperactivity disorder (ADHD) is a neurodevelopmental disorder with an estimated prevalence in children and adolescents of 7.2% according to a systematic review published recently [1]. ADHD is characterized by difficulties in maintaining sustained attention, hyperactivity and acting on impulse. Among the consequences of this disorder are a higher percentage of accidents, higher rates of school dropouts, or a greater probability of having addiction problems [2,3]. Moreover, ADHD increases mortality between two and eight times in children, adolescents, and adults when it is not properly treated [4]. Accordingly, an accurate and early diagnosis of ADHD is fundamental to improve its poor prognosis.

Usually, ADHD diagnosis is based on the judgment of the health professional using a clinical history often supported by scales filled out by caregivers and/or teachers. Therefore, the ADHD diagnosis depends primarily on health professionals’ expertise and the caregiver/teacher’s observational skills [5]. Several experts have criticized this way of diagnosing ADHD as they indicate that it tends to be subjective on both clinicians’ and caregivers’ side [6,7]. For instance, a recent study has shown that a group of 473 psychotherapists, specialized in children and adolescents, committed more than 15% of false positives and about 20% of false negatives in identifying this disorder through medical records [8]. In another study, Schultz and Evans showed that young female teachers tended to provide higher scores than older male teachers [9]. Finally, it has also been shown that parents’ and caregivers’ evaluations can be influenced by their mood [10].

In addition to the observational capabilities of the medical professionals and caregivers, the assessment’s accuracy may also be influenced by various weaknesses associated with the use of questionnaires. As an example, the veracity of the responses may not be guaranteed. In the case of ADHD, some of the reasons for which patients tend to exaggerate or attenuate their symptoms are: to justify academic failure, to access to stimulant drugs, to obtain certain social/academic benefits, or to refuse that they have the disorder [11,12]. Furthermore, limited accuracy is sometimes obtained with questionnaires and scales [13].

In order to surpass these limitations, in recent years, some authors have proposed to analyze patients’ behavior while performing a computerized task [14]. As will be shown below in the bibliographic review, these works are based on the go-no-go paradigm. Unlike these existing tests, this article proposes a proper video game aiming at accurately assessing the severity of inattention in patients with ADHD. In this game genre, the player has to make a running avatar avoid different obstacles that are in its way. Specifically, in our game, the avatar will have to jump to avoid falling into different gaps that are in its way. Our hypothesis is that children diagnosed with ADHD will commit more omissions and perform a greater number of jumps near the gap as a result of distractions. The popularity of this game’s type makes patients feel familiar and therefore increases its ecological validity. Examples of some games of this genre can be easily found and freely downloaded (i.e., temple run, subway surfer or jet pack joy ride [15]). Another difference compared with some computerized tasks requiring virtual reality equipment is that the proposed video game can be executed on any standard desktop or mobile device. This allows the evaluations to be performed at zero cost during the consultation or even telematically.

The rest of the article is structured as follows. Section 2 reviews the existing computerized task for assessing ADHD. Then, in Section 3, the materials and methods are described. These include the description of the sample, the developed video game, and the statistical methods. In Section 4, the results obtained in an experiment aiming to evaluate the proposed video game are presented. The article concludes in Section 5 with a discussion of the found benefits of the proposed video game and pointing out future lines of research.

## 2. Bibliographic Review

As mentioned above, a significant number of computerized tasks have been developed in recent years aimed at identifying patients with ADHD [14]. A common characteristic is that all of them are continuous performance tests (CPTs) based on the go-no-go paradigm [16].

Some of these works extend the traditional CPT to make it more similar to a video game. For example, Berger, Slobodin, and Cassuto proposed the MOXO-CPT in which the letters were replaced by cartoons, and visual and auditory distracters were added [17]. Concisely, the target was a child’s face, and the distractors were five animals including a duck with a similar color and shape to the target. The researchers observed that the measures collected in a traditional CPT (number of correct responses, reaction time, omissions, and commissions) consistently distinguished between children with ADHD and their unaffected peers. Specifically, they reported an area under the curve (AUC) of 0.96 as a precision measure. The AUC is often used to evaluate the performance of a classifier. It takes values between 0.5 and 1, where 0.5 suggests discrimination not better than a random guess. Although there is no criterion for determining when an AUC is good, some authors consider that a value higher than 0.9 suggests outstanding discrimination [18]. However, a weakness of this work is that the researchers did not split the data into training and testing sets in their analysis. This may cause the reported results to be better than those obtained in a new sample. In another work, Shaw, Grayson, and Lewis conducted a similar study using images of Pokemon [19]. However, these authors found no significant difference between children with ADHD and controls (AUC close to 0.5). This discordance can be explained by the fact that they tried to identify children with ADHD by taking into account only the number of commissions which was the least discriminating measure in the work of Berger, Sloboding, and Cassuto.

A different extension was proposed by Keller et al. [5]. In their game “Groundskeeper”, inspired by the popular game “Whac-A-Mole”, the keyboard was replaced by Sifteos cubes. These cubes are able to digitally display different images and interact with each other by proximity. The patient had to move a cube with the image of a mallet towards any of the other three cubes when the image of a gopher appeared and not bring it closer when the image of birds, a rabbit or the groundkeeper showed up. The novelty of this work was not only that the human–computer interaction was tangible, but also the high number of predictors it collected. These predictors were analyzed by several recent machine learning techniques such as decision trees, boosting, or random forest. Although their results were quite accurate in discriminating children with ADHD from controls, the researchers observed that they were not able to improve the predictive ability obtained either by the standard CPT or by the Conners’ Brief Rating Scale.

Later, these authors replicated the study by replacing the previous predictive techniques with a logistic regression [20]. In this study, “Groundskeeper” achieved better results than both CPT and the Conners’ Brief Rating Scale. However, the same as the previously commented works, this study had the weakness of not having analyzed the data by means of cross-validation or a repeated validation, which reduces the generalization of the results.

Another work in which the interaction between the participant and the computer was carried out through movement was developed by Delgado-Gomez et al. [21,22]. In their study, patients reacted to the stimuli that appear in the CPT by raising their dominant hand instead of pressing the space bar of the keyboard. Three-dimensional positions of the dominant hand were captured 30 times per second using a Kinect camera. In this way, they were able to identify events that could not be captured on a standard CPT, such as when the participant started the reaction but stopped it before pressing the space bar. The authors reported that they obtained more accurate assessments of the participants’ impulsiveness than with the Conners’ CPT.

In order to incorporate ecological validity into the assessment, Rizzo et al., proposed the use of virtual reality [23]. In 2006, Rizzo et al., developed the virtual classroom, a three-dimensional virtual environment that mimics a classroom. In their study, the participants performed a CPT in which the stimuli appeared on the blackboard of the virtual classroom [24]. In this study, which included eight children diagnosed with ADHD and 10 controls, the authors noted that children with ADHD had slower hit reaction times, higher reaction time variability, and made more omissions and commissions errors. Using a sample of 10 children with ADHD and 10 controls, Parsons et al., replicated the study conducted by Rizzo and his colleges obtaining similar results [25]. The novelty of their work is that the authors compared the measures obtained in the virtual classroom with those obtained in the Conners’ CPT-II, observing a significant correlation in the omissions and commissions [26]. These results were later verified by Diaz-Orueta et al., in a sample of 52 children diagnosed with ADHD [27]. In addition, Bioulac et al., observed that, in a sample of 36 children, the performance degradation over the course of the virtual classroom test was similar to the obtained at CPT-II [28]. Recently, Areces et al., found out that, in the virtual classroom, the number of commissions and the motor activity, measured through the head mounted display, was lower in the inattentive subtype than in the hyperactive subtype [29].

The following section describes the proposed raccoon runner game. Unlike the previous computerized tasks, the proposed video game does not follow a go-no-go paradigm. It is a standard video game that most children are familiar with, which increases the ecological validity of the test. It also has the advantage of not needing specific hardware. This considerably reduces its cost and allows it to run on personal computers, tablets, or mobile devices, allowing remote evaluations.

## 3. Materials and Methods

### 3.1. Participants

A group composed of 32 children (29 males) referred to the Child and Adolescent Psychiatry Unit of the Department of Psychiatry at Fundación Jiménez Díaz Hospital (Madrid, Spain) and diagnosed with ADHD according to DSM-5 criteria participated in the study [30]. All participants were receiving medication. Among the participants, 10 were diagnosed as inattentive type while the remaining 22 were diagnosed as combined type. The mean and standard deviation of the age was 12.46 and 3.01, respectively. The minimum age was 8, and the maximum was 16.

### 3.2. Running Raccon Game

**Figure 1:**
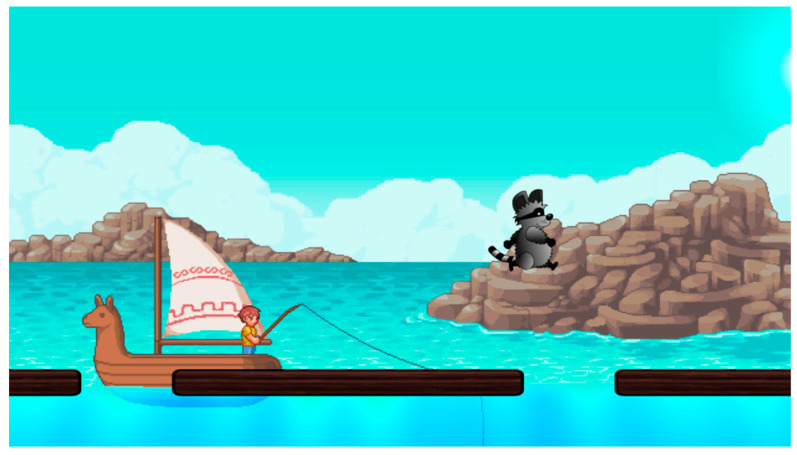
Running raccoon game screenshot.

The running raccoon game is a video game based on the genre of infinity runners in which a raccoon must jump several gaps before reaching the goal. The game was implemented using the widely used Unity 3D game engine [31]. Figure 1 shows a screenshot of the game.

In detail, the raccoon has to jump 180 gaps which are grouped into 18 blocks. Each block is identified by the raccoon’s speed, the trunk length, and gap length. The length of the trunk and the speed of the avatar define the inter stimuli (IS) time, which is approximately 1.5, 2.5, and 3.5 s while the gap’s width defines the difficulty of the jump. The settings of the different blocks are shown in Table 1.

### 3.3. Inatention SWAN Rating Subscale

The Attention-Deficit/Hyperactivity Disorder Symptoms and Normal Behavior rating scale (SWAN) is a parent/caregiver report inventory developed for screening ADHD [32]. It extends the 18-item ADHD rating scale-IV by increasing the number of possible responses for each item from four to seven [33]. On the SWAN scale, each item is scored from −3 to +3 (below average to above average), where 0 is “normal”. A strong internal consistency and moderate test–retest reliability has been reported [34]. The Swan scale is composed of two subscales. The first nine items are related to inattention, while the last nine items are related to hyperactivity and impulsivity. In this article, the inattention subscale is used.

During the experiment, a trained psychiatrist accompanied each of the patients while they conducted the task. While each child was performing the test, the corresponding caregiver or legal tutor filled the inattention subscale of the SWAN scale. The average score obtained was −7.1 and the standard deviation was 10.7.

### 3.4. Statistical Analysis

The predictors that are widely used in the literature were computed. These are the median and interquartile range (IQR) of the recorded distances along with the number of omissions for each participant. The recorded distance is the distance from the jump point to the beginning of the gap, while the number of omissions represents the number of times the participant did not jump. Pearson’s correlation was calculated for each of these measures and the score obtained in the inattention subscale of the SWAN scale. In addition, a multiple regression analysis was conducted to analyze if these predictors are independent.

### 3.5. Ethics Procedures

Caregivers were required to sign their informed consent after been explained the test in detail. The consent form and the study protocol were reviewed and approved by the Institutional Review Board of Fundación Jiménez Díaz Hospital of Madrid. During the experiment, a trained psychiatrist accompanied each of the patients while they conducted the task. While each child was performing the test, the corresponding caregiver filled the inattention subscale of the SWAN scale.

## 4. Results

The first column in Table 2 shows the correlations (and *p*-value) of the median and the interquartile range of the jump distances and the number of omissions made by each participant with respect to the score obtained by them on the inattention subscale of the SWAN scale. Columns 2 to 4 in Table 2 display the correlation considering only the jumps where the time between stimuli is 1.5, 2.5, and 3.5, respectively. Whenever the time between stimuli is less than 2 s, the correlations are no longer significant, and when it is greater than 2 s, they increase.

A relevant aspect to investigate is whether these widely used predictors are independent. Table 3 shows the T statistics and the *p*-values associated with the coefficients of the variables included in the different possible linear regression models. The fact that the variables are significant in the simple linear regression models and no longer in the multiple models shows the collinearity of these variables.

This result makes sense since, for example, the shorter the jump distance, the more likely it is to commit an omission (the raccoon automatically jumps when it collides with the edge of the gap). For this reason, the following analyses are performed using only the median of the jump distances.

**Figure 2:**
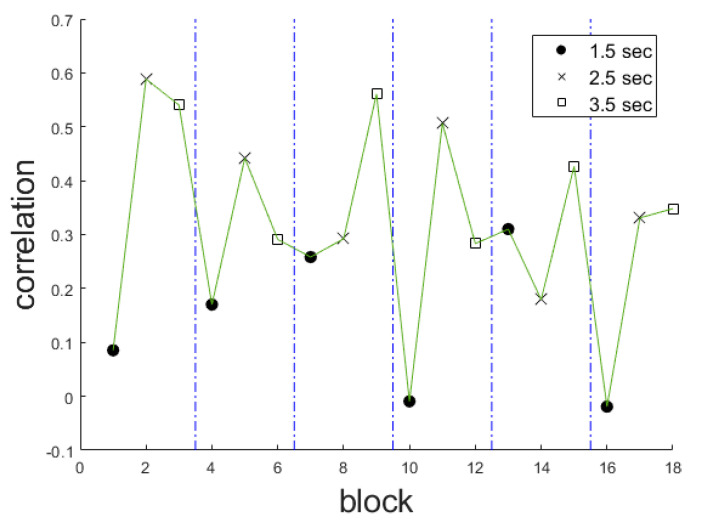
Correlations between the median jump distance and the inattention subscale score.

Another important aspect to investigate is whether the value of these correlations depends on the length of the test. That is, whether the correlations obtained with these three measures are higher at the beginning or the end of the test. Figure 2 shows the correlation obtained between the median of the jump distances and the participant’s inattention score for each of the 18 blocks defined above. It can be observed that these correlations do not depend on the stage of the test. However, the correlations were higher when the time between stimuli is long (2.5 or 3.5 s) than when the time between stimuli is shorter (1.5 s).

**Figure 3:**
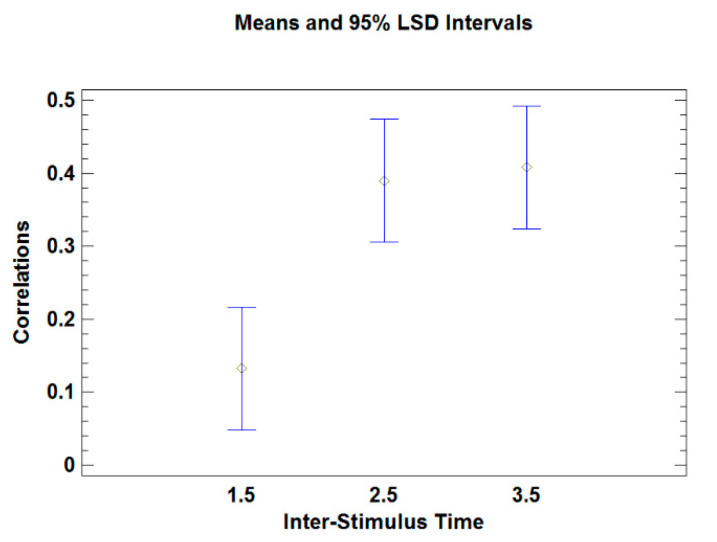
Means comparison.

In order to verify the last statement, an ANOVA test was conducted in which the dependent variable is the correlation and the factor is the time between stimuli. The analysis showed that at least one of the means was significantly different from the others (*p*-value = 0.005). To get a better understanding, Figure 3 displays the mean plot. In addition, to determine which means are statistically significantly different from the others a multiple range test was conducted. A Bonferroni multiple comparison procedure identified that the mean of the correlations obtained when the interstimulus time was 2.5 or 3.5 s are statistically significant different from those obtained when the interstimulus time was 1.5 s. It did not identify a significant difference between the mean of the correlations obtained for the 2.5 and 3.5 interstimulus times.

To assess the performance of the proposed game, a repeated validation experiment was performed [35,36]. To do this, the previous available data were divided into two disjoint sets. These two sets are usually called training and validation sets. The training set is used to estimate the parameters of the model, while the evaluation set is used to assess it. The key point is that the validation set is not used when the model is trained and therefore it plays the role of a new sample. For this purpose, 75% of the observations (*n* = 24) were used to estimate the parameters of the linear regression model, while the remaining 25% were used to evaluate it. The linear regression model was built with three predictors. These were the medians calculated using the jump distances of each of the three blocks with similar interstimulus times. For each observation in the validation set, the inattention of the associated participant was estimated by the built linear regression model using the three participants’ predictors. The correlation of these estimates with the scores obtained by those participants in the SWAN inattention subscale was calculated. To obtain more significant results, 10,000 repetitions were performed and the average of the obtained correlations was calculated. The mean correlation obtained was 0.53.

## 5. Discussion and Conclusions

In the present study an adaptation of a traditional video game of the infinity runner genre has been proposed to assess the degree of inattention of children with ADHD. This work differs from previous studies in two aspects. Firstly, with the exception of the work of Shaw, Grayson and Lewis [19], it is a genuine video game instead of a computerized task based on the go-no-go paradigm. Secondly, our article assesses the severity of inattention and does not focus on discriminating against children with ADHD from controls.

Our results suggest that the number of times the avatar does not jump, as well as the median and interquartile range of the jump distances, show a significant correlation with the severity of patients’ inattention. In addition, this correlation tends to be greater when the time between stimuli increases. This could be explained because when the time between stimuli is short, the patient is immersed in the game, whereas whenever this time is longer, ADHD patients have difficulty maintaining the attention. This finding suggests giving more importance to the jumps in which the interstimulus time is longer (i.e., those in which the time between stimuli is greater than two seconds).

The proposed methodology has several advantages. First of all, unlike the existing methods that last more than 15 min, the developed test takes approximately seven minutes. Furthermore, our results indicate that a shorter test could be sufficient to accurately evaluate ADHD. This feature makes it especially attractive in clinical environments where time is scarce. Second, the test does not require complicated or expensive hardware such as virtual reality equipment. A standard computer has been used in this work, but devices such as tablets or mobile devices could also be used. The limitations of our study are the sample size, the use of a single assessment scale and the absence of a healthy control group. Future studies with larger samples and administered with different assessment scales will help to confirm our pilot results. In addition, the availability of a control group will also allow us to analyze whether the proposed game is capable of discriminating children with ADHD from the participants in the control group.

In conclusion, the results obtained open up new lines of research. Firstly, to find out what is the optimal length and configuration (time between stimuli) of the test. Secondly, since the game can be run on any device, to analyze the possibility of performing the test remotely. More importantly, our study indicates to use other video game genres (graphic adventures, strategy, puzzles, etc.) as diagnostic tools for ADHD or any other mental disorders.

## Figures and Tables

**Table 1:** Length of the trunk and gap (in Unity units), speed of the raccoon (in units per second) and the Inter Stimuli time (in seconds) for each of the blocks.

Block	Trunk	Gap	Speed	IS Time	Block	Trunk	Gap	Speed	IS Time
**B1**	8	2	5	1.6	**B10**	11	3	7	1.6
**B2**	13	2	5	2.6	**B11**	18	3	7	2.6
**B3**	18	2	5	3.6	**B12**	25	3	7	3.6
**B4**	7	3	5	1.4	**B13**	9.5	4.5	7	1.4
**B5**	12	3	5	2.4	**B14**	16.5	4.5	7	2.4
**B6**	17	3	5	3.4	**B15**	23.5	4.5	7	3.4
**B7**	6	4	5	1.2	**B16**	8	6	7	1.2
**B8**	11	4	5	2.2	**B17**	15	6	7	2.2
**B9**	16	4	5	3.2	**B18**	22	6	7	3.2

For each jump, it is recorded whether the participant jumped or not, and if so, the distance from the jump point to the beginning of the gap is also recorded. As discussed in the Introduction, we hypothesize that the distance from the jump point to the gap will be related to the attention process in the sense that inattentive children will jump closer to the border as a result of distractions.

**Table 2:** Correlations (*p*-value) of the three predictors obtained with respect to the inattention subscale both for the complete test and for the blocks with equal time between stimuli.

	All	1.5 s	2.5 s	3.5 s
**Median**	0.48 (<0.01)	0.23 (0.21)	0.55 (<0.01)	0.52 (<0.01)
**IQR**	0.43 (0.01)	0.11 (0.56)	0.40 (0.02)	0.41 (0.02)
**Omissions**	−0.39 (0.03)	−0.21 (0.25)	−0.49 (<0.01)	−0.41 (0.02)

IQR: interquartile range.

**Table 3:** T statistics and *p*-values associated with the coefficients of the variables included in the different linear regressions.

Variables Included in the Model	Median	IQR	Omission
**Median**	2.98 (0.005)	-	-
**IQR**	-	2.59 (0.014)	-
**Omission**	-	-	−2.54 (0.016)
**Median+IQR**	1.32 (0.196)	0.18 (0.855)	-
**Median+Omission**	1.86 (0.073)	-	−0.73 (0.468)
**IQR+Omission**	-	1.79 (0.083)	−1.43 (0.161)
**Median+IQR+Omissions**	0.62 (0.524)	0.42 (0.678)	−0.81 (0.42)

## References

[1] Thomas R., Sanders S., Doust J., Beller E., Glasziou P. (2015). Prevalence of Attention-Deficit/Hyperactivity Disorder: A Systematic Review and Meta-analysis. Pediatrics.

[2] Harpin V. (2005). The effect of ADHD on the life of an individual, their family, and community from preschool to adult life. Arch. Dis. Child..

[3] Elkins I.J., McGue M., Iacono W.G. (2007). Prospective Effects of Attention-Deficit/Hyperactivity Disorder, Conduct Disorder, and Sex on Adolescent Substance Use and Abuse. Arch. Gen. Psychiatry.

[4] Dalsgaard S., Østergaard S.D., Leckman J.F., Mortensen P.B., Pedersen M.G. (2015). Mortality in children, adolescents, and adults with attention deficit hyperactivity disorder: A nationwide cohort study. Lancet.

[5] Heller M.D., Roots K., Srivastava S., Schumann J., Srivastava J., Hale T.S. (2013). A Machine Learning-Based Analysis of Game Data for Attention Deficit Hyperactivity Disorder Assessment. Games Health.

[6] Edwards M.C., Gardner E.S., Chelonis J.J., Schulz E.G., Flake R.A., Diaz P.F. (2007). Estimates of the Validity and Utility of the Conners’ Continuous Performance Test in the Assessment of Inattentive and/or Hyperactive-Impulsive Behaviors in Children. J. Abnorm. Child Psychol..

[7] Pellegrini S., Murphy M., Lovett E. (2020). The QbTest for ADHD assessment: Impact and implementation in Child and Adolescent Mental Health Services. Child. Youth Serv. Rev..

[8] Bruchmüller K., Margraf J., Schneider S. (2012). Is ADHD diagnosed in accord with diagnostic criteria? Overdiagnosis and influence of client gender on diagnosis. J. Consult. Clin. Psychol..

[9] Schultz B.K., Evans S.W. (2012). Sources of Bias in Teacher Ratings of Adolescents with ADHD. J. Educ. Dev. Psychol..

[10] Chi T.C., Hinshaw S.P. (2002). Mother-child relationships of children with ADHD: The role of maternal depressive symptoms and depression-related distortions. J. Abnorm. Child Psychol..

[11] Frazier T.W., Frazier A., Busch R.M., Kerwood M.A., Demaree H.A. (2008). Detection of simulated ADHD and reading disorder using symptom validity measures. Arch. Clin. Neuropsychol..

[12] Sansone R.A., Sansone L.A. (2011). Faking Attention Deficit Hyperactivity Disorder. Innov. Clin. Neurosci..

[13] Basco M.R., Bostic J.Q., Davies D., Rush A.J., Witte B., Hendrickse W., Barnett V. (2000). Methods to Improve Diagnostic Accuracy in a Community Mental Health Setting. Am. J. Psychiatry.

[14] Calvo I.P., Jiang-Lin L.K., Girela-Serrano B., Delgado-Gomez D., Navarro-Jimenez R., Baca-Garcia E., Porras-Segovia A. (2020). Video games for the assessment and treatment of attention-deficit/hyperactivity disorder: A systematic review. Eur. Child Adolesc. Psychiatry.

[15] Dietrich B. Forget the battery, let’s play games!. Proceedings of the IEEE 12th Symposium on Embedded Systems for Real-time Multimedia.

[16] Trommer B.L., Hoeppner J.-A.B., Lorber R., Armstrong K.J. (1988). The Go—No-Go paradigm in attention deficit disorder. Ann. Neurol..

[17] Berger I., Slobodin O., Cassuto H. (2017). Usefulness and Validity of Continuous Performance Tests in the Diagnosis of Attention-Deficit Hyperactivity Disorder Children. Arch. Clin. Neuropsychol..

[18] Hosmer D.W., Lemeshow S., Sturdivant R.X. (2013). Applied Logistic Regression.

[19] Shaw R., Grayson A., Lewis V. (2005). Inhibition, ADHD, and Computer Games: The Inhibitory Performance of Children with ADHD on Computerized Tasks and Games. J. Atten. Disord..

[20] Faraone S.V., Newcorn J.H., Antshel K.M., Adler L., Roots K., Heller M. (2016). The Groundskeeper Gaming Platform as a Diagnostic Tool for Attention-Deficit/Hyperactivity Disorder: Sensitivity, Specificity, and Relation to Other Measures. J. Child Adolesc. Psychopharmacol..

[21] Delgado-Gómez D., Carmona-Vázquez C., Bayona S., Ardoy-Cuadros J., Aguado D., Baca-Garcia E., Lopez-Castroman J. (2015). Improving impulsivity assessment using movement recognition: A pilot study. Behav. Res. Methods.

[22] Delgado-Gómez D., Calvo I.P., Masó-Besga A.E., Vallejo-Oñate S., Tello I.B., Duarte E.A., Varela C.V., Carballo J., Baca-Garcia E., Baron D. (2017). Microsoft Kinect-based Continuous Performance Test: An Objective Attention Deficit Hyperactivity Disorder Assessment. J. Med. Internet Res..

[23] Rizzo A., Buckwalter J., Bowerly T., Van Der Zaag C., Humphrey L., Neumann U., Chua C., Kyriakakis C., Van Rooyen A., Sisemore D. (2000). The Virtual Classroom: A Virtual Reality Environment for the Assessment and Rehabilitation of Attention Deficits. CyberPsychol. Behav..

[24] Rizzo A., Bowerly T., Buckwalter J.G., Klimchuk D., Mitura R., Parsons T.D. (2009). A Virtual Reality Scenario for All Seasons: The Virtual Classroom. CNS Spectr..

[25] Parsons T.D., Bowerly T., Buckwalter J.G., Rizzo A.A. (2007). A Controlled Clinical Comparison of Attention Performance in Children with ADHD in a Virtual Reality Classroom Compared to Standard Neuropsychological Methods. Child Neuropsychol..

[26] Conners C.K., Sitarenios G. (2011). Conners’ Continuous Performance Test (CPT).

[27] Díaz-Orueta U., Garcia-López C., Crespo-Eguílaz N., Sanchez-Carpintero R., Climent G., Narbona J. (2013). AULA virtual reality test as an attention measure: Convergent validity with Conners’ Continuous Performance Test. Child Neuropsychol..

[28] Bioulac S., Lallemand S., Rizzo A., Philip P., Fabrigoule C., Bouvard M.P. (2012). Impact of time on task on ADHD patient’s performances in a virtual classroom. Eur. J. Paediatr. Neurol..

[29] Areces D., Rodríguez C., García T., Cueli M., González-Castro P. (2016). Efficacy of a Continuous Performance Test Based on Virtual Reality in the Diagnosis of ADHD and Its Clinical Presentations. J. Atten. Disord..

[30] Guze S.B. (1995). Diagnostic and Statistical Manual of Mental Disorders, 4th ed. (DSM-IV). Am. J. Psychiatry.

[31] Unity 3D. https://unity.com.

[32] Swanson J.M., Schuck S., Porter M.M., Carlson C., Hartman C.A., Sergeant J.A., Clevenger W., Wasdell M., McCleary R., Lakes K. (2012). Categorical and Dimensional Definitions and Evaluations of Symptoms of ADHD: History of the SNAP and the SWAN Rating Scales. Int. J. Educ. Psychol. Assess..

[33] Hay D.A., Bennett K.S., Levy F., Sergeant J., Swanson J. (2007). A Twin Study of Attention-Deficit/Hyperactivity Disorder Dimensions Rated by the Strengths and Weaknesses of ADHD-Symptoms and Normal-Behavior (SWAN) Scale. Biol. Psychiatry.

[34] Lakes K.D., Swanson J.M., Riggs M. (2012). The reliability and validity of the English and Spanish strengths and weaknesses of ADHD and normal behavior rating scales in a preschool sample: Continuum measures of hyperactivity and inattention. J. Atten. Disord..

[35] James G., Witten D., Hastie T., Tibshirani R. (2013). An Introduction to Statistical Learning.

[36] Dubitzky W., Granzow M., Berrar D.P. (2007). Fundamentals of Data Mining in Genomics and Proteomics.

